# Molecular Study from the Signaling Pathways of Four Potential *asthma triggers*: AKT1, MAPK13, STAT1, and TLR4

**DOI:** 10.3390/ijms26136240

**Published:** 2025-06-28

**Authors:** Lucía Cremades-Jimeno, María López-Ramos, Rubén Fernández-Santamaría, María Ángeles De Pedro, Ignacio Mahillo, Cristina Rosales-Ariza, José María Olaguibel, Victoria del Pozo, María Luisa Caballero, Juan Alberto Luna-Porta, Santiago Quirce, Blanca Barroso, Diana Betancor, Marcela Valverde-Monge, Joaquín Sastre, Selene Baos, Blanca Cárdaba

**Affiliations:** 1 Immunology Department, IIS-Fundación Jiménez Díaz-UAM, 28040 Madrid, Spain; lucia.cremades@quironsalud.es (L.C.-J.); mlr3041996@gmail.com (M.L.-R.); ruben.fsantamaria@quironsalud.es (R.F.-S.); mpedrm1@gmail.com (M.Á.D.P.); cristina.rosales@quironsalud.es (C.R.-A.); vpozo@fjd.es (V.d.P.); selenebmuniz@gmail.com (S.B.); 2 Biostatistics and Epidemiology Unit, University Hospital Fundación Jiménez Díaz, 28040 Madrid, Spain; imahillo@fjd.es; 3 Allergology Service, University Hospital of Navarra, 31008 Pamplona, Spain; jm.olaguibel.rivera@navarra.es; 4 Ciber de Enfermedades Respiratorias (CIBERES), 28029 Madrid, Spain; mlcsoto@hotmail.com (M.L.C.); juanalberto.luna@salud.madrid.org (J.A.L.-P.); squirce@gmail.com (S.Q.); jsastre@fjd.es (J.S.); 5 Department of Allergy, La Paz University Hospital, IdiPAZ, 28029 Madrid, Spain; 6 Allergy Department, University Hospital Fundación Jiménez Díaz, 28040 Madrid, Spain; blanca.barroso@quironsalud.es (B.B.); diana13_b@hotmail.com (D.B.); marcela.valverde@quironsalud.es (M.V.-M.)

**Keywords:** asthma, biomarkers, allergic asthma (AA), nonallergic asthma (NA), AKT1, MAPK13, STAT1, TLR4, peripheral blood mononuclear cells (PBMCs), gene expression, signaling pathways

## Abstract

Asthma is a chronic and heterogeneous inflammatory airway disease with diverse clinical endotypes and limited curative treatment options. Recent systems biology analyses identified four potential molecular *triggers*—AKT1, MAPK13, STAT1, and TLR4—as candidate regulators of asthma-associated signaling pathways. This study aimed to validate the expression of these four proteins and their downstream signaling elements in peripheral blood mononuclear cells (PBMCs) from patients with allergic asthma (AA), nonallergic asthma (NA), and healthy controls (HC), to explore their potential as biomarkers or therapeutic targets. For that, PBMC samples were collected from 45 AA patients, 17 NA patients, and 15 HC subjects. Gene and protein expression of AKT1, MAPK13, STAT1, and TLR4 were quantified using RT-qPCR and Western blotting. Expression patterns were compared across groups and stratified by asthma severity. Correlations with clinical parameters (FEV1, FVC, FeNO, IgE, eosinophil counts) and treatment regimens were also assessed. All four target genes showed significantly reduced expression in asthma patients compared to controls (*p* < 0.001), with the most marked downregulation in NA patients. At the protein level, MAPK13 and TLR4 showed significant differential expression. Stratification by severity revealed a stepwise reduction in gene expression in AA patients, correlating with disease severity, whereas NA patients showed uniformly low expression regardless of severity. Multiple pathway-related genes, including *RELA*, *SMAD3*, *NFATC1*, and *ALOX5*, were also downregulated, particularly in NA patients. Notably, differential correlations were observed between gene expression and lung function parameters in AA vs. NA groups. In conclusion, this study supports the potential involvement of AKT1, MAPK13, STAT1, and TLR4 in asthma pathogenesis and highlights differences between allergic and nonallergic asthma at the molecular level. These proteins and their associated pathways may serve as future targets for biomarker development or endotype-specific therapies. Further studies in larger and more diverse cohorts, including functional validation, are warranted.

## 1. Introduction

Highly prevalent chronic respiratory diseases, such as asthma, remain a priority healthcare challenge, despite significant progress in recent years. It is defined by the Global Initiative on Asthma (GINA) as a heterogeneous disease characterized by chronic airway inflammation [1]. Asthmatic patients have recurrent respiratory symptoms including shortness of breath, wheezing, chest tightness, coughing, and fatigue which limits expiratory airflow [2]. Generally, asthma is characterized by unpredictable attacks and exacerbations, which require continuous patient monitoring and frequent medication adjustments and changes, but in no case those treatments are curative. Clinically, asthma is associated with inflammation and a progressive decline in lung function. Allergic mechanisms have been implicated in 50–80% of asthmatic patients and in approximately 50% of severe asthmatics. This is one of the reasons why, traditionally, asthma has been associated with type 2 (T2) respiratory inflammation, characterized by elevated levels of IgE, eosinophils, and certain cytokines such as IL-4, IL-5, IL-13, and IL-9, canonically associated with allergic responses. However, not all patients present this type of inflammation. Although conventional therapies control the symptoms in a significant proportion of patients, precise diagnostic tools capable of predicting the highly heterogeneous nature of the disease, including its development, progression, severity, and adequate response to treatment, are still lacking. Advances in understanding the pathobiological mechanisms have enabled the clinical implementation of new biological therapies for the most severe cases with poorly controlled symptoms using conventional therapies. These therapies are primarily aimed at reducing type 2 inflammation (monoclonal antibodies against IgE, IL-4, IL-5, IL-13, TSLP, and the IL-5 receptor) [3,4,5]. Although the results with these biological agents are promising, there is some variability in relation to their efficacy, partly due to the high heterogeneity within T2 inflammation [6]. Besides, the nontype 2 asthma endotype (with a prevalence of neutrophilic inflammation or a neutrophil/eosinophil combination) is less characterized [7]. Therefore, no therapies with biologics against non-T2 inflammation have been developed to date. In addition, these therapies are targeted to the control of the symptoms, and the final goal of asthma treatment is to develop a disease-modifying therapy approach. This could be achieved through the development of therapies targeting specific inflammatory pathways in the pathogenesis of asthma, probably yet to be discovered [5,8,9].

The need to define new subtypes of asthma has been reclaimed for clinicians and researchers, and many different approaches could be used, as was recently summarized [10]. Clinical features, laboratory parameters, and -omic techniques are crucial in identifying asthma endotypes for better understanding of the immunological mechanism and treatment. Refining current endotypes and discovering new ones through multi-omics can improve asthma outcomes by informing treatment beyond the T2-centric focus [11].

In this context, we recently [12] defined at a molecular level three respiratory pathologies (allergic and nonallergic asthma and respiratory allergy) using systems biology, in means of mathematical models able to simulate each disease. With those models, we analyzed the ability of 94 proteins to modulate the activation of an important number of effector proteins of these pathologies. The objective of this strategy was to identify the proteins of interest whose modulation could promote the activation of the highest number of proteins involved in these three diseases, especially in asthma. With this, we described four proteins, AKT1, MAPK13, STAT1, and TLR4, that together were theoretically capable of activating a significant percentage of disease-defining proteins. These proteins were named *asthma triggers*, since their activation could trigger the asthmatic symptoms in allergic and nonallergic asthmatic patients. The four theoretical *asthma triggers* defined play essential roles in numerous cellular processes. Serine/threonine kinase 1 (AKT1) is an important regulator of cell growth, survival, and metabolism. It has been associated with airway hyperactivity, airway inflammation, and airway remodeling [13,14]. Mitogen-activated protein kinase 13 (MAPK13) is the p38δ isoform of the p38MAPK family, also called stress-activated protein kinases, that respond to inflammatory and environmental physical insults. The p38 MAPK family (α, β, γ, and δ) consists of highly conserved proline-directed serine-threonine protein kinases that are activated in response to a number of inflammatory signals, but they differ in their tissue distribution, regulation of kinase activation, and subsequent phosphorylation of downstream substrates [15]. The p38 MAPK pathway is crucial in inflammation, cell death and cell proliferation, and therefore has been implicated in many cellular events relevant to asthma pathophysiology being propose as good candidate for asthma treatment [16]. The Toll-like receptor 4 (TLR4) is a transmembrane protein receptor that is critical for proper activation of innate immunity. TLRs are induced by pathogen-associated molecular patterns (PAMPs), which are found in diverse pathogenic organisms and absent from the host [17]. TLRs are located on and within immune and nonimmune cells and are critical for initiating adaptive immune responses, playing essential roles to maintain immune homeostasis [18,19,20]. Finally, signal transducer and activator of transcription 1 (STAT1) is one of the seven members, which regulate, via the Janus Kinases (JAK)/STAT pathway, essential processes like cell proliferation, differentiation, apoptosis, and immune function in response to extracellular signals such as cytokines and growth factors [17].

Summarizing, in this study, we experimentally validated the expression of these four candidate *asthma triggers* and their signaling cascades in peripheral blood mononuclear cells to find experimental evidences that support the involvement of these signaling axes in asthma endotypes as a first point to further functional dissection as potential targets for endotype-specific therapeutic interventions. The final purpose is trying to define potential new therapeutic targets that could be relevant in the management of patients’ outcomes, helping the future treatment of asthma.

## 2. Results

### 2.1. Subjects

The study population was composed of 15 healthy control subjects (HC), 45 allergic asthmatic (AA) and 17 nonallergic asthmatic (NA) patients. The demographic and clinical characteristics of this population are presented in Table 1.

According to demographic characteristics, we observed a significantly higher mean age in the AA patients (48.02 ± 12 y.o; *p* < 0.0001) and NA patients (54.88 ± 11 y.o; *p* < 0.0001) than in the HC subjects (32.87 ± 10.8 y.o), without statistically significant differences between both asthma groups. The AA group had a higher presence of women (73.3%). Finally, most of the AA patients were non-smokers (55.6%), while most of the NA patients were ex-smokers (52.9%). About 11% of patients were still smokers in both asthma groups.

Patients were classified according to having AA (skin test positive and/or specific IgE against to at least one airborne allergen) or NA, patients who did not present any sensitization to aeroallergens. AA patients showed a positive skin test to pollens (36.5%), mites (29.5%), mites and pollens (2%), pet epithelial (25%), and other allergens (7%).

Both groups were also classified according to their asthma severity in mild, moderate, and severe patients defined by the Spanish Guide for Asthma Management (GEMA) [21]. While in the AA group, the ratio between the three severities was almost equal; in the NA patients the moderate diagnosis was higher (47%) than mild (29.5%) or severe (23.5%). The lung function was good in all the groups, with percentages of forced expiratory volumen in 1 s (FEV1) and forced vital capacity (FVC) above 85% without statistically significant differences between asthmatic patients and HC. As expected, the AA group presented the highest total IgE (tIgE) mean sera levels (436.71 ± 493.1 kU/L) compared to the HC (63.55 ± 94.7 kU/L; *p* = 0.0002) and the NA group (159.95 ± 173.2 kU/L; *p* = 0.04). The NA group also presented a significantly higher mean sera levels of total IgE than the HC subjects (*p* = 0.05). Finally, the fractional exhaled nitric oxide (FeNO) was higher in both asthma groups (38.4 ± 30.7 ppb in AA and 32.38 ± 27 ppb in NA), compared to HC (11.1 ± 5.9 ppb), but only the AA patients showed statistically significant differences compared to HC group (*p* = 0.006). No differences between both asthma groups were found.

Blood and sputum analyses were performed in asthmatic groups to better characterize the inflammatory profile of the patients, based on the presence of eosinophils and neutrophils. The eosinophil and neutrophil levels in peripheral blood and percentages in sputum were slightly higher in the NA patients, but without reaching statistical significance. However, the presence of eosinophils and neutrophils in sputum allowed us to classify the inflammatory profile of patients based on the predominant cell type as follows: eosinophilic (≥3% eosinophils), neutrophilic (≥70% neutrophils), mixed (≥3% eosinophils and ≥70% neutrophils), and paucigranulocytic (<3% eosinophils and <70% neutrophils). In both groups, the paucigranulocytic and eosinophilic were the predominant profiles, with similar percentages, while the neutrophilic profile was observed in a higher percentage of patients in the NA group (17.6% vs. 4.4% in AA). Sputum and blood eosinophils and neutrophils did not show any correlation in any of the groups.

Finally, according to the asthma treatment, most of the patients (>90%) were treated with inhaled corticosteroids and long-acting β2-agonists, and almost half of them were also treated with short-acting bronchodilators. Also, approximately 30% of AA patients received some form of specific immunotherapy. A total of 17.6% of NA patients were also receiving some form of biological treatment, higher in AA patients (40%), specifically, omalizumab or mepolizumab.

### 2.2. The Four Potential Gene Triggers Show Different Gene and Protein Expression in Asthma Patients than in the Healthy Subjects

**Figure 1:**
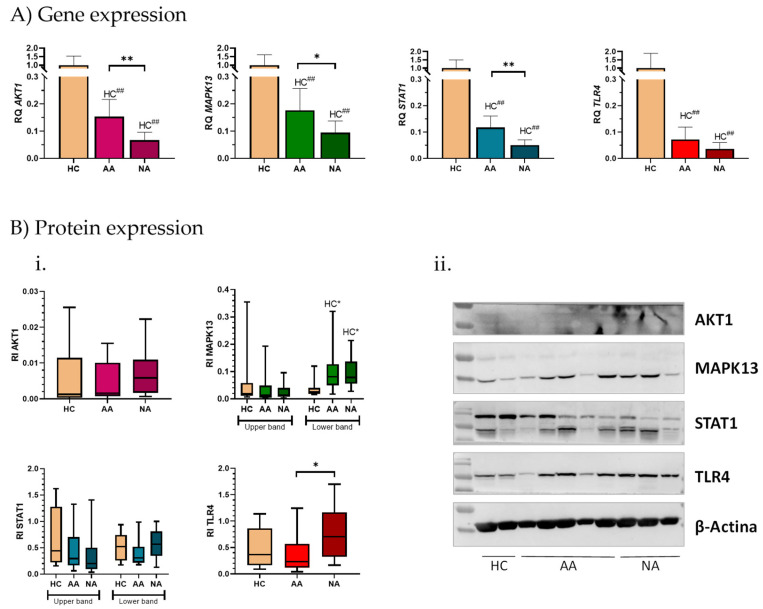
Differential gene and protein expression results of AKT1, MAPK13, STAT1, and TLR4 in PBMCs samples. (**A**) The relative expression of each gene is represented as the mean relative quantification (RQ) in each asthma group compared to healthy controls. Error bars represent the standard error. (**B**) (**i**) Protein expression results are displayed in a box-and-whisker plot, which shows the relative intensity (RI) of each band detected by Western blot and the β-Actin band in each sample. (**ii**) A representative example of a Western blot including two control subjects (HC), five allergic asthmatic patients (AA), and three nonallergic asthmatic patients (NA) is shown. Statistically significant differences between groups, according to the nonparametric Mann–Whitney test, are indicated: HC* and HC^##^ (*p* < 0.05 and *p* < 0.001, respectively) for statistically significant differences between the control group and the indicated clinical group; * and ** (*p* < 0.05 and *p* < 0.01, respectively) for statistically significant differences between the two clinical groups indicated by the horizontal bar.

Gene and protein expression of the four potential asthma triggers, AKT1, MAPK13, STAT1, and TLR4, was analyzed in PBMC samples obtained from AA, NA, and HC (Figure 1).

At the gene level, both asthmatic patient groups showed a statistically significant decrease in the expression of all genes (*p* < 0.001) compared to the HC healthy control group (Figure 1A). Furthermore, in all genes the expression was lower in the NA than in the AA group, these differences being statistically significant in *AKT1* (NA = 0.067, AA = 0.154; *p* = 0.008), *MAPK13* (NA = 0.095, AA = 0.177; *p* = 0.021), and *STAT1* (NA = 0.051, AA = 0.117; *p* = 0.007), but without reaching the statistical signification in *TLR4* (NA = 0.036, AA = 0.072; *p* = 0.06).

Minor differences were observed in the protein expression (Figure 1(Bi)). From the representative image of the profile observed for each protein (Figure 1(Bii)) in the Western blot, one faint 63 kDa band in AKT1 was observed; two 52 and 43 kDa bands in MAPK13; two 130 and 95 kDa in STAT1; and one 100 kDa band in TLR4. The expression of these proteins was very heterogeneous between the different subjects.

Firstly, a statistically significant decrease was found in the lower band of MAPK13 in HC (RI = 0.037), compared to asthmatic patients (AA RI = 0.103 and *p* = 0.01; NA RI = 0.099 and *p* = 0.01). On the other hand, TLR4 showed a significantly higher expression in the NA than in the AA group (AA RI = 0.364 and NA RI =0.765, *p* = 0.02). It is worth highlighting the different behavior of the two strips detected in MAPK13, where the upper seems to have a higher expression in the HC, while the lower band shows a higher expression in the asthmatic patients. Regarding AKT1, a slight increase in protein expression was observed in the NA group compared to HC and AA patients. In STAT1, the lower band also shows this behavior, but in the upper band, on the contrary, the highest expression was observed in HC.

### 2.3. A Deeper Analysis Showed Differences in the Results Obtained for Each Clinical Group When Patients Were Segregated by Severity

**Figure 2:**
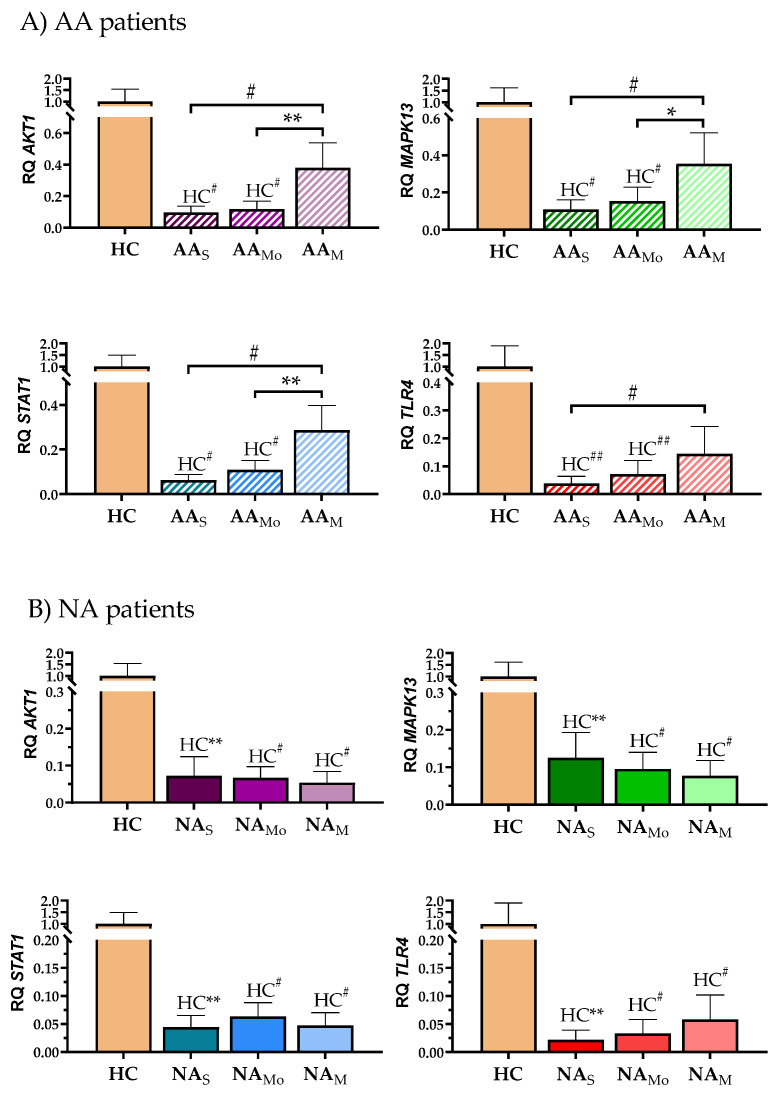
Differential gene expression of potential *asthma triggers* according to asthma severity in (**A**) allergic asthmatic (AA) patients and (**B**) nonallergic asthmatic (NA) patients. The relative expression of each gene is represented as the mean relative quantification (RQ) in each asthma group compared to healthy controls. Error bars represent the standard error. The gene expression was compared among severities and with healthy control (HC) subjects by non-parametric Mann–Whitney test, using the R program, and differences were considered significant with adjusted *p*-values under 0.05. Statistically significant differences between HC and the indicated severity subgroup are shown as HC**, HC^#^, and HC^##^ (*p* ≤ 0.01, *p* ≤ 0.005, and *p* ≤ 0.001, respectively). Statistically significant differences among indicated subgroups are shown as *, **, and ^#^ (*p* ≤ 0.05, *p* ≤ 0.01, and *p* ≤ 0.001, respectively). AA_S_, AA_Mo_, AA_M_: severe, moderate, and mild allergic asthmatic patients, respectively; and NA_S_, NA_Mo_, NA_M_: severe, moderate, and mild nonallergic asthmatic patients, respectively.

We analyzed the gene expression of the four *triggers* by segregating asthma patients according to their severity (Figure 2). In the AA group, the lowest gene expression of the four genes was shown in severe patients and it increased as the severity was reduced (Figure 2A). These differences were statistically significant when HC subjects were compared with severe or moderate, but not with mild patients. In AA group, we also observed differences between severities. Interestingly, *AKT1*, *MAPK13*, and *STAT1* gene expression showed a similar behavior, with statistically significant differences between the results obtained in the mild patients and the moderate and severe patients, but in TLR4 only severe and mild patients showed significant differences. By contrast, in the NA group all the severity subgroups showed a significant decrease in the expression of the four studied genes compared with the HC subjects. No differences between severities were observed (Figure 2B).

### 2.4. The Expression of the Four Potential Triggers Showed a Good Gene Correlation Between Them, but Worse at a Protein Level, with Differences Among Clinical Groups

Correlation between the expression of the four potential asthma triggers was assessed. The results (Table 2) showed good correlations among the gene expression of the four triggers in all the groups of the study, AKT1 and MAPK13 being the best correlated in HC group (Rs = 0.97; *p* < 0.001) and AKT1 and STAT1 in AA and NA patients (Rs = 0.92; *p* < 0.001 and Rs = 0.94; *p* < 0.001, respectively). TLR4 was the gene that showed the lower correlation with the rest of the genes in all the groups. However, according to protein expression, few statistically significant correlations were found, TLR4 being the protein that showed the highest correlations: with AKT1 in HC (Rs = 0.89; *p* = 0.001), with STAT1 in AA (Rs = 0.84; *p* < 0.001), and inversely with MAPK13 in NA (Rs = −0.62; *p* = 0.05).

### 2.5. The Four Potential Triggers Showed Different Correlation with Lung Functional Parameters Depending of the Group Studied

To determine the clinical implication of these four asthma triggers, a correlation analysis between their expression and clinical parameters (peripheral eosinophils/mL, Feno, tIgE levels, and lung function) was performed (Table 3). This analysis showed different correlations in AA than NA, drawing attention how even the direction of the correlations was different in both groups: while in AA all the correlations were negative, in NA all were positive, except for the peripheral blood eosinophils that showed the opposite behavior. Specifically, at a protein level the AA group showed the most significant negative correlations with the protein expression of STAT1 (with FEV1) and TLR4 (with FEV1 and FVC). At a gene level, we observed correlations between *MAPK13* and *STAT1* expression and FEV1 and FVC; and between *TLR4* expression and the ratio FEV1/FVC. In contrast, NA patients showed the best positive correlations with protein expression of TLR4 (with FEV1 and FVC), and STAT1 expression with FVC. At a gene level, *MAPK13* expression showed correlation with the ratio FEV/FVC and *STAT1* expression correlated with FVC. Peripheral blood eosinophils correlated positively with *STAT1* gene expression in AA and negatively with STAT1 protein expression in NA. The rest of the clinical parameters did not show statistically significant results.

### 2.6. Analysis of the Signaling Pathways of the Triggers Proteins Show a Significant Gene Expression Decrease in Asthma Groups Compared to HC Subjects

Our next step was the analysis of the gene expression of the main elements defined in the theoretical signaling pathways of each of the four *asthma triggers*, according to previous results [12], to experimentally corroborate their involvement in those pathways. From the 29 genes studied (detailed in Material and Methods), five, *CCL11*, *CCL17*, *IL12B*, *IL17B*, and *MUC5B*, were not detected in the samples analyzed. The remaining 24 genes were grouped by pathway and by their theoretical assignment as previously established (link nodes or mechanistic proteins). Table 4 shows the differential gene expression of those specific genes defined in endotype-specific pathways (AA or NA) in whole groups and severity subgroups, compared to HC.

Both asthma groups showed a statistically significant decrease in all the genes analyzed compared to HC subjects, as happened with the asthma triggers. However, the analysis according to asthma severity showed a different behavior in the expression of these genes depending on the asthma phenotype. In AA patients, the gene expression differences with HC decreased in the less severe phenotypes of the disease to the point that in the comparison between mild AA patients and HC subjects, only IL13RA1 showed statistically significant differences (Table 4A). In contrast, the NA patients showed very similar gene expression in all the severity subgroups compared with HC subjects (Table 4B): statistically significant differences in all genes except CXCL8 in the three severity subgroups, and CDH1 and TLR5 in severe patients.

The expression of these genes was also compared between the different severities within each asthma group. While the differential gene expression between the severity of NA subgroups did not show any statistically significant result, in AA group all genes showed statistically significant differences between mild and severe patients, fewer genes between mild and moderate patients (*AKT1* (adjusted *p* = 0.005), *RELA* (adjusted *p* = 0.006), *MAPK13* (adjusted *p* = 0.02), *NFATC1* (adjusted *p* = 0.002), *STAT1* (adjusted *p* = 0.002), *CCL5* (adjusted *p* = 0.004), *SMAD3* (adjusted *p* = 0.008), and in the four genes of the proteins with implication in the mechanisms: *IL4R* (adjusted *p* =0.004), *ALOX5* (adjusted *p* = 0.005), *BAX* (adjusted *p* = 0.003), and *TGFB1* (adjusted *p* = 0.03). No gene showed significant differences between moderate and severe patients. This highlights the different behavior of these genes in both asthma endotypes.

### 2.7. NA Patients Have Lower Gene Expression than AA Patients on the Common Genes to Both Signaling Pathways

The differences in gene expression between AA vs. NA of those common genes in the signaling pathways were analyzed. This was done in total groups and after segregating the patients by asthma severity. Firstly, all genes showed a lower expression in the NA group, this decrease being statistically significant in all genes except *TLR4* (Table 5). However, when both groups were analyzed according to patients’ severity, no significant differences were observed in either severe or moderate subgroups, but in mild patients all genes except *TLR2* and *TLR4* were significantly decreased in the NA group (adjusted *p* = 0.003 for *AKT1*, *RELA*, *STAT1* and *ALOX5*; adjusted *p* = 0.004 for *SMAD3* and *NFATC1*; adjusted *p* = 0.005 for *MAPK13*).

## 3. Discussion

Asthma is a chronic disease caused by an imbalance in the immune system, characterized by inflammation, remodeling, and airway hyperreactivity, resulting from a complex, variable network of molecules that differ within each patient and between patients. Certain networks are repetitive among asthma patients and link to clinical expression, gene–environment interaction, and inflammatory cell profiles, offering new diagnostic and therapeutic strategies for specific endotypes. Besides, emerging evidence indicates that asthma pathogenesis may involve both airway inflammation and inflammation-independent mechanisms [22,23,24,25,26]. Unraveling the molecular mechanisms underlying requires multiple efforts and has been the challenge of many researchers for years [11,17]. Cell signaling pathways are crucial in inflammatory diseases playing a central role in intercellular communication. -Omics technology and bioinformatic advances enable pathway delineation, and the present work is an example. Here, we have analyzed theoretical signaling pathways related to four *asthma trigger* proteins, AKT1, MAPK13, STAT1, and TLR4, defined by previous systems biology study as proteins able to modify the activation of many effector proteins of respiratory diseases [12]. The final purpose of this work was to confirm the deregulation of these pathways in asthma disease and to define new targets that could be relevant as a starting point to future treatments.

All the four potential *asthma triggers* studied have an essential role in numerous cellular processes. AKT1 is involved in cell proliferation, differentiation, and survival by signal transduction through the PI3K/Akt/mTOR axis [13,27]. MAPK13 integrates various signals and regulates several cellular processes such as proliferation, differentiation, transcription regulation, and development. It can be activated by inflammatory cytokines and cellular stress. The MAPKs signaling pathway is crucial for immune response and is targeted in treatments for inflammatory disorders [15,16]. However, the role of p38MAPK usually is studied as global protein family, and the δ isoform is one of the 4 isoforms with less specific information. MAPK13 has been linked to cytokine production, cell migration, inflammasome activation, and T cell activation at the immune level [28,29]. STAT1 is a transcription factor that has been classically implicated in the Th1 inflammatory response due to its role in the response to IFNγ [30,31]. STAT1 activation has been implicated in the pathogenesis of allergic diseases [32,33]. Very recently it has been described that IL-9 induction was restrained by IFN-γ/STAT1 and IL-10, pointing to a new role of this pathway in the modulation of asthma disease [34].

Finally, TLR4 is a transmembrane receptor of the TLR family, with a fundamental role in immune response to extracellular stimuli [35,36], classically involved in the Th2 response, although under certain conditions, it can induce the Th1 response [37]. To maintain immune homeostasis, the activation of innate immune cells requires TLR signaling molecules. The strength of TLR signaling does determine the occurrence of allergic reactions or its absence. Strong TLR signals are protective against allergic airway disease, while low airway amounts of TLR ligands cause airway sensitization and Th2-type immunity [18,19,20]. In bronchial asthma, it was shown that TLR4 activation of macrophages produces cytokines that affect immune Th1/Th2 balance [38].

The activation pathways of these four proteins are closely related, with TLR4 as a central element in the regulation of the rest of *triggers*. Its activation and subsequent binding to different adaptors, such as MyD88 or TRIFF, promote the activation of the PI3K/AKT axis, as well as the p38MAPK pathway, with multiple effects on immune response [39,40,41]. Within the p38MAPK pathway, it has been shown that the inhibition of p38γ and δ leads to an absence of response to lipopolysaccharides (LPS), a molecule typically related to TLR4 signaling [42]. There is also a relationship between the activation of TLR4 and STAT1, dependent and independent of IFNγ signaling [43,44]. This cytokine has also been linked to STAT1 activation through the PI3K/AKT axis [45]. In view of this, it is expected that variations in these proteins jointly affect the development of asthma, although their concrete implication in asthma has never been addressed. Independently, AKT1 has been linked to the Th2 response in vivo and in vitro models [46,47], as well as to the response to treatments [48,49], although there are discrepancies regarding its involvement in airway inflammation [50,51]. Regarding STAT1, attempts have been made to analyze its influence on desensitization to ICS without obtaining conclusive results [52,53]. Multiple evidences suggest that toll-like receptors may be associated with the atypical stimulation of immune responses, contributing to the chronic inflammation seen in asthma. TLRs can affect epithelial and immune cell function in asthma. Of note, TLR4 is essential for Th17-driven neutrophilic airway inflammation and neutrophil recruitment [54,55]. Numerous authors have linked TLR4 to allergic sensitization and asthma, both by promoting and reducing the inflammatory response [48,56,57], and its increase in the airways of asthmatic patients has been linked to a worse prognosis [58].

Based on this information, it is not surprising that these proteins were related to molecular motifs involved in immune system related to asthma and that their combined *trigger* potential was defined by systems biology [12]. Therefore, in this work these *triggers* were analyzed in PBMCs from asthmatic patients and healthy subjects. First, the gene and protein expression of these four proteins were analyzed (Figure 1). Patients with asthma showed significantly lower gene expression compared to healthy controls. The gene decrease was more pronounced in NA patients compared to AA patients, with significant differences between both groups except for *TLR4*. Also, the relationship with severity was analyzed segregating the patients according to their asthma severity (Figure 2). The severe AA patients showed the lowest gene expression of the four genes, although all the severity subgroups showed gene decrease compared to the HC subjects. These differences were statistically significant when HC subjects were compared to severe or moderate patients, but not with mild patients (Figure 2A). There were also differences between severities. *AKT1*, *MAPK13*, and *STAT1* were significantly decreased in moderate and severe patients compared to mild patients. *TLR4* was significantly decreased in the severe patients compared to mild patients. However, the NA patients showed a significant decrease in the expression of the four studied genes in all the severity subgroups, compared with the HC subjects, and no differences between severities were observed (Figure 2B). However, at protein level only MAPK13 and TLR4 showed statistically significant differences among groups. In contrast, the protein expression of AKT1 was very similar in all groups, with a slight increase observed in the NA group, and STAT1 showed reduced protein levels in asthmatic patients, but without statistically significant differences, contrasting with previous observations [59]. Some works had related the expression of this gene in response to viral infections, an asthma trigger in some nonallergic patients [60,61], which could explain these results. Regarding MAPK13, two bands were detected by Western blot (Figure 1) that did not show the same behavior, so it would be of great interest to delve deeper into the possible post-translational modifications of this protein and its function in asthma. The lower band was the only protein with significantly higher expression in asthma patients compared to healthy subjects. This aligns with MAPK13’s known role in inflammatory mechanisms. However, this different behavior of gene and protein expression suggests a complex control of this signaling pathway. Methylation is one potential regulatory mechanism for this gene, as observed by other groups [62,63].

Finally, TLR4 showed the highest protein expression in NA patients, being significantly higher than that observed in AA patients. The expression of TLR4 was lower in monocytes and dendritic cells, but not in CD4+ T cells, of asthmatic patients who had been previously defined [64]. In addition, the regulation of TLR4 activation is strictly controlled by internalization mechanisms, and abnormal trafficking of this protein has already been linked to different diseases [65,66]. Therefore, in-depth studies of its expression in different cell types, as well as its activation, are necessary to understand the role of TLR4 in asthma.

Having in mind the relationship that these four proteins have, gene and protein correlation analysis between them was performed, corroborating this relationship (Table 2). There were statistically significant correlations among the gene expression of the four triggering in all the groups of the study, *AKT1* and *MAPK13* being the best correlated in HC group, and *AKT1* and *STAT1* in asthmatic patients. However, according to protein correlations, few statistically significant correlations were found, TLR4 being the protein that showed the highest correlations with AKT1 in HC, with STAT1 in AA, and with MAPK13 in NA, this last one being a negative correlation.

Finally, to determine the clinical associations of these four *asthma triggers*, a correlation analysis between their gene/protein expression and clinical parameters was performed. Results of the lung functional analysis showed different correlations in AA and NA (Table 3); remarkably, in AA all the correlations found were negative, which means that the lowest lung function comes with the highest ΔCt and protein expression, while in NA the correlations were positive. These results could not strongly demonstrate the involvement of these triggers in lung function, because the significant correlations with functional parameters were weak in many cases but highlight the possible different functions of these proteins in the different inflammation stages of asthmatic patients.

Searching for a possible explanation of these results, we analyzed the relationship of the gene and protein expression of the four *asthma triggers* and medications taken (Supplementary Figures S1 and S2). We found higher differences among patients with and without treatment in the gene expression than in the protein expression. Specifically, *AKT1*, *MAPK13,* and *STAT1* gene expression were significantly decreased in patients taking long-acting β-antagonists (LABA). It was previously described that LABAs and p38MAPK inhibitors reverse the corticosteroid insensitivity of IL-8 in airway smooth muscle cells of COPD [67]. TLR4 gene expression was decreased in patients using bronchodilators and biologics (Omalizumab and Mepalizumab). In contrast, specific immunotherapy increased the gene-expression of the four *asthma triggers*. According to protein expression, patients taking biologics (Omalizumab and Mepalizumab) showed STAT1 expression significantly decreased and TLR4 expression significantly increased. Allergen-specific immunotherapy (AIT), in contrast, significantly decreased TLR4 protein expression. There is scarce information from the relationship of these proteins and treatments, but it recently has been demonstrated in an allergic asthma rat model the role of AIT inhibiting the HMGB1/TLR4/NF-κB signaling, in agreement with our data [68]. Those data must be confirmed but point to different molecular mechanisms underlying the different treatment response and underscore the relevance of keeping in mind the treatment of the patients in molecular studies.

To conclude, the gene expression of the proteins involved in the signaling pathways of the four *asthma trigger* proteins was analyzed (Table 4), finding a decrease of all of them in both asthma groups compared to HC, and in those elements common to the signaling pathways defined for AA and NA (*RELA*; *SMAD3*; *NFATC1*, *TLR2* and *ALOX5*), this decrease was greater in the NA patient group (Table 5), in line with what was observed in the four *triggers* (Table 1). Curiously, in mild AA patients, a tendency to normalize these values was observed, which did not occur in NA patients. Of these common elements, some stand out, such as *RELA* or NF-kB subunit, a master transcriptional regulator of the committed epithelial–mesenchymal transition in airway epithelial cells [69]; *SMAD3*, a protein that functions in the transforming growth factor-beta signaling pathway and transmits signals from the cell surface to the nucleus, regulating gene activity and cell proliferation [70]; or *NFATC1* or nuclear factor of activated T cells, a critical transcription factor for immune regulation with an essential role in T and myeloid cells [71]. It should be noted that the effector protein of the four pathways common to both asthma phenotypes, *ALOX5* or 5-lipooxygenase (5-LO), an enzyme that uses Arachidonic Acid to synthesize Leukotrines (LTs), which induce airway spam, barely showed differences between the two asthma groups in severe and moderate patients but did in mild patients. This lipoxygenase has previously been related to asthma, mainly, to the response to treatment of patients [72,73] and could be an interesting potential therapeutic target in the treatment of asthma, allergic and nonallergic.

This study has several limitations, as the number of NA patients recruited that made it difficult to extrapolate solid conclusions, mainly when the patients were segregated by severities. Also, NA patients showed a statistically significantly higher BMI than healthy control subjects (27.5 ± 4.8 vs. 23.6 ± 1.3; *p* < 0.05), but were not classified as obese based on BMI (25.0–29.9). There is an interesting asthma phenotype, obesity-associated asthma, whose study has shown specific molecular characteristics, as recently described [74,75]. Although our patients did not present obesity, overweight could be related to molecular changes that should be investigated further in relation to the objectives of our study. Also, we have studied PBMCs, a mix of cells, and this could be a limitation for having clear conclusions. Studies with sorter populations and epigenetic analyses are needed.

Thus, a bigger study population and longitudinal or functional validation of findings is mandatory to clarify the results obtained in this study, mainly from NA patients. Further, the results shown here are related to peripheral and immune information, so a study of the relevance of these triggers in the lung (as target-organ of asthma) is essential, as their role could be different in this organ. Overall, our results show a decrease in the gene expression of the four *asthma triggers* studied and the molecules involved in their signaling pathways. This unexpected result must be validated in functional assays (increasing/decreasing our targets) for understanding how this gene decrease is associated with functional aspects of asthma disease and trying to find key elements not delimitated in the present study.

In conclusion, this work is to our knowledge the first assessing the expression of these four *asthma triggers* in combination and trying to find potential therapeutic targets. Overall, our results highlight the importance of these pathways in asthma, highlighting several common molecular elements (*RELA*, *SMAD3*, *NFATC1*, *TLR2,* and *ALOX5*) that were downregulated in AA and NA in the most severe phenotypes, opening possibilities in the search of new treatments useful for all asthmatic patients. These findings provide molecular evidence that position AKT1, MAPK13, STAT1, and TLR4 as central nodes in the pathophysiology of asthma, justifying future functional studies to delimitate their specific roles and attempt new therapeutic interventions.

## 4. Materials and Methods

### 4.1. Study Design: Subjects

The study population comprised 62 unrelated asthmatic patients, 45 with allergic asthma (AA) and 17 with nonallergic asthma (NA), who followed the selection criteria: aged 18–75 years and diagnosed with asthma at least 1 year before inclusion. The samples were selected from the biobank of the cohort from the MEGA [76] project. Subjects were diagnosed in the allergy and pulmonology departments of the *Fundación Jiménez Díaz* Hospital in Madrid, and in the allergy departments of *La Paz* Hospital in Madrid, and *Complejo Hospitalario de Navarra* in Pamplona. Samples were processed in each hospital. As a control group, 15 healthy subjects (HC) were recruited in the allergy departments of *Fundación Jiménez Díaz* Hospital in Madrid, Policlinic Hospital in Seville, and *San Cecilio* University Hospital in Granada, Spain; and samples were processed in the immunology department of the Health Research Institute-*Fundación Jiménez Díaz-UAM* (IIS-FJD-UAM) in Madrid.

For all subjects, tests of pulmonary function were carried out by determining the predicted percentage of forced vital capacity (%FVC) and the forced expiratory volume in 1 s (%FEV1). AA patients were asthmatic and sensitized to at least one airborne allergen, while NA patients were diagnosed with asthma disease but had a negative skin prick test. In both groups, the asthma severity was diagnosed as severe, moderate, or mild according to the Spanish Guidelines for the Management of Asthma (GEMA) [21]. The HC group was made up of healthy subjects with no history of respiratory diseases or allergic symptoms, and were negative to skin prick test against a panel of common allergens, including mites (*Dermatophagoides pteronyssinus*, *Dermatophagoides farinae*, and *Lepidoglyphus destructor*), epithelia (cat and dog), cockroaches (*Blatella orientalis* and *Blatella germanica*), pollens (*Cypress*, *banana shadow*, olive, mixture of grasses, *Artemisia*, *Parietaria*, and *Salsola*), and fungi (*Alternaria*, *Cladosporium*, *Aspergillus*, and *Penicillium*).

Written informed consent was obtained from each subject in accordance with the Declaration of Helsinki. Ethical approval for the study was obtained from the research ethics committee of the IIS-FJD-UAM.

### 4.2. Isolation of Peripheral Blood Mononuclear Cells, RNA, and Protein Extraction

Peripheral blood samples (PBMCs) were obtained from all the subjects included in the study. PBMCs were isolated from heparinized blood by gradient centrifugation using Lymphoprep (Comercial Rafer, Zaragoza, Spain) following the manufacturer’s instructions, under sterile conditions using endotoxin-free reagents. RNA and protein were extracted from PBMCs (10^6^ cells) using the trizol method (TRItidy G, PanReac AppliChem, Darmstadt, Germany), following the manufacturers’ instructions. RNA quantification and purity (A260/A280 > 1.7) was checked by spectrophotometry using a spectrophotometer (Nanodrop ND-1000, Bonsái Technologies Group, Madrid, Spain), while protein was quantified with the Pierce BCA Protein Assay Kit (ThermoFisher Scientific, Waltham, MA, USA), following the manufacturer instructions.

### 4.3. Gene Selection and Differential Expression Analysis by RT-qPCR

A total of 29 genes were selected according to previous results of our group, based on their classification within the signaling pathways of the four theoretically defined asthma triggers (Table 6). Thus, the four triggers were studied, *AKT1*, *MAPK13*, *STAT1,* and *TLR4,* along with genes classified as mechanistic in at least one asthma endotype (*ALOX5*, *BAX*, *CCL5*, *CCL11*, *IL4R*, *IL17A*, *MUC5B*, *PTGER2*, *TGFB1)* and link nodes between both groups of genes (*CCL17*, *CDH1*, *COL5A1*, *CXCR3*, *IL8*, *IL10*, *IL12A*, *IL12B*, *IL13RA1*, *MMP9*, *NFATC1*, *RELA*, *SMAD3*, *TLR2*, *TLR5*, *VCAN)*. The 18S gene expression was used as endogenous control.

Gene expression was analyzed in the Scientific Park of Cantoblanco (Madrid, Spain) by quantitative real-time PCR, using TaqMan Gene Expression System (Applied Biosystems, Foster City, CA, USA) in 384-well microfluidic cards. Briefly, 1.5 µg of RNA from each subject were converted to cDNA trough reverse transcription, using “High-capacity RNA to cDNA kit” (Applied Biosystems). Then, real time PCR was performed using Taqman Gene Expression and the HT7900 System (Applied Biosystems) with 40 amplification cycles. The results were analyzed with the SDS software v2.3 (Applied Biosystems, Foster City, CA, USA). Gene expression of the 29 selected genes was analyzed in duplicates, and expression of *18S* gene was used as a reference, using the Thermofisher Scientific predesigned TaqMan Gene expression assays indicated in Table 6.

Finally, Relative Quantification (RQ) values were calculated according to the cycle threshold (Ct) method, with the gene expression represented as 2^−ΔΔCt^, where ΔΔCt = (ΔCt_clinical group_) − (ΔCt_control group_) and ΔCt = (Ct_study gene_) − (Ct_18S_).

### 4.4. Differential Protein Expression Analysis by Western Blot

Protein expression was analyzed by Western blot using the Invitrogen Western Breeze^®^ Chemiluminescent Western Blot Immunodetection Kit (Life Technologies, Carlsbad, CA, USA) as previously described [77]. The primary antibodies and conditions used to detect the four triggering proteins were the mouse IgG anti-human AKT1 or TLR4 (diluted 1:200 and 1:500, respectively) paired with the HRP-coupled secondary antibody m-IgG Fc (1:2500 and 1:5000, respectively) from Santa Cruz Biotechnology; and the rabbit IgG anti-human MAPK13 or STAT1 (both diluted 1:1000) from Cell Signaling and Thermo Fisher, respectively, paired with the ready to use anti-rabbit secondary antibody from the Invitrogen kit coupled with the AP enzyme. The visualization was performed using chemiluminescent specific substrates and the ImageQuant LAS 4000 luminescent image analyzer (GE Healthcare Life Science, Little Chalfont, Buckinghamshire, UK). Quantification results of specific protein bands were relativized to rabbit IgG anti-human β-Actin (dilution 1:1000; Cell Signaling) expression, through the equation RI (relative intensity) = specific protein intensity/b-actin protein intensity.

### 4.5. Statistical Analysis

Multiple comparisons for the differential gene expression analysis were performed, using the R program, with the obtained ΔCt values. Thus, gene expression was compared between clinical groups and severity subgroups through the ANOVA test for multiple comparisons, using Benjamini–Hochberg for posterior corrections. Also, simple comparisons between the differential protein expressions were performed with the non-parametric Mann–Whitney test, using the relative intensity data. The correlation analysis of the expression of each gene or protein with the other triggers, as well as their gene expression with functional parameters, was performed with the nonparametric Spearman test. A strong correlation was considered when the r coefficient (Rs) value was ≥0.80, moderate when it was between the values 0.50–0.79, and weak when r < 0.50.

## 5. Conclusions

AKT1, MAPK13, STAT1, and TLR4 and their associated pathways are deregulated at peripheral level in asthmatic patients compared with healthy control subjects. The results provide molecular evidence that position these four proteins as central nodes in the pathophysiology of asthma, justifying future functional studies to delimitate their specific roles and attempt new therapeutic interventions.

## Figures and Tables

**Table 1:** Demographic and clinical characteristics of the study population.

	HC	AA	NA
N	15	45	17
Age (y.o), mean ± SD	32.87 ± 10.8	48.02 ± 12 ^##^	54.88 ± 11 ^##^
Gender, N (%)			
Male	7 (46.7)	12 (26.7)	7 (41.2)
Female	8 (53.3)	33 (73.3)	10 (58.8)
Smoking habit, N (%)			
Smoker		5 (11.1)	2 (11.8)
Ex- Smoker		13 (28.9)	9 (52.9)
No Smoker		25 (55.6)	6 (35.3)
ND	15 (100)	2 (4.4)	0 (0)
BMI (Kg/m^2^), mean ± SD	23.6 ± 1.3	25.87 ± 4.5	27.5 ± 4.8 *
Asthma severity, N (%)			
Severe	-	17 (37.8)	4 (23.5)
Moderate	-	14 (31.1)	8 (47)
Mild	-	14 (31.1)	5 (29.5)
Spirometry, mean ± SD			
FEV_1_ (%)	100.82 ± 7.9	87.74 ± 20	96.89 ± 21.8
FVC (%)	104 ± 6.9	98.88 ± 18.7	110.1 ± 20.6
FEV_1_/FVC	82.53 ± 2.2	77.09 ± 9.8	81.78 ± 14.4
Fe_NO_ (ppb), mean ± SD	11.1 ± 5.9	38.4 ± 30.7 **	32.38 ± 27
Total IgE (IU/mL), mean ± SD	63.55 ± 94.7	436.71 ± 493.1 ^#,†^	159.95 ± 173.2 *
Blood cells			
Eosinophils (cells/mm^3^), mean ± SD	280 ± 168.7	324.67 ± 206.1	424.8 ± 205
Neutrophils (cells/mm^3^), mean ± SD	ND	3823 ± 1574.6	4438.33 ± 1172.6
Sputum cells			
Eosinophils (%), mean ± SD	ND	9.6 ± 17.4	16 ± 27.9
≥3% eosinophils, N (%)	ND	10 (22.2)	5 (29.4)
Neutrophils (%), mean ± SD	ND	29.4 ± 24	40 ± 30.4
≥70% neutrophils, N (%)	ND	4 (8.8)	3 (17.6)
Cellular profile, N (%)			
Eosinophilic	ND	9 (20)	5 (29.4)
Neutrophilic	ND	2 (4.4)	3 (17.7)
Mixed	ND	1 (2.2)	0 (0)
Paucigranulocyte	ND	13 (28.9)	4 (23.5)
ND	ND	19 (42.2)	5 (29.4)
Treatment, N (%)			
Inhaled Corticosteroids (ICS)	-	43 (95.5)	17 (100)
Systemic Corticosteroids	-	1 (2.2)	2 (11.8)
Long-term β2-Agonists (LABA)	-	39 (86.7)	17 (100)
Short-term anticholinergics	-	1 (2.2)	1 (5.9)
Long-term anticholinergics	-	9 (2)	2 (11.8)
Leukotriens receptor agonists	-	12 (26.7)	3 (17.7)
Short-term bronchodilators	-	18 (40)	7 (41.2)
Specific immunotherapy	-	12 (26.7)	0 (0)
Biologics (Omaluzimab, Mepaluzimab)	-	14 (31.1), 4 (8.8)	2 (11.8), 1(5.9)

HC: healthy control subjects. AA: allergic asthmatic patients. NA: nonallergic asthmatic patients. SD: Standard deviation. ND: No data. BMI: Body mass index. FEV1: Forced expiratory volume in 1 s. FVC: Forced vital capacity. FeNO: Fractional exhaled nitric oxide (ppb). Total IgE units: IU/mL (International Units per mL). Statistically significant diferences between clinical groups and healthy control are shown as *, **, ^#^, ^##^ for values *p* < 0.05, *p* < 0.01, *p* < 0.001, and *p* < 0.0001, respectively. Statistically significant diferences between groups and NA are shown as ^†^ for *p* < 0.05.

**Table 2:** Analysis of correlation among triggering gene (cursive) and protein expression by clinical groups.

(A) Healthy control				
	AKT1	MAPK13	STAT1	TLR4
AKT1		***Rs = 0.97; p < 0.001***	***Rs = 0.73; p = 0.002***	***Rs = 0.60; p = 0.02***
MAPK13	**Rs = 0.82; *p* = 0.004**		***Rs = 0.69; p = 0.005***	***Rs = 0.64; p = 0.01***
STAT1	Rs = 0.51; *p* = ns	Rs = 0.57; *p* = ns		***Rs = 0.55; p = 0.04***
TLR4	**Rs = 0.89; *p* = 0.001**	Rs = −0.19; *p* = ns	**Rs = 0.74; *p* = 0.02**	
(B) Allergic asthmatic patients				
	AKT1	MAPK13	STAT1	TLR4
AKT1		***Rs = 0.86; p < 0.001***	***Rs = 0.92; p < 0.001***	***Rs = 0.71; p < 0.001***
MAPK13	Rs = −0.41; *p* = ns		***Rs = 0.83; p < 0.001***	***Rs = 0.60; p < 0.001***
STAT1	Rs = 0.10; *p* = ns	**Rs = 0.74; *p* = 0.01**		***Rs = 0.75; p < 0.001***
TLR4	Rs = −0.47; *p* = ns	Rs = 0.59; *p* = ns	**Rs = 0.84; *p* < 0.001**	
(C) Nonallergic asthmatic patients				
	AKT1	MAPK13	STAT1	TLR4
AKT1		***Rs = 0.92; p < 0.001***	***Rs = 0.94; p < 0.001***	***Rs = 0.53; p = 0.04***
MAPK13	Rs = −0.53; *p* = ns		***Rs = 0.83; p < 0.001***	***Rs = 0.53; p = 0.04***
STAT1	Rs = −0.35; *p* = ns	Rs = −0.36; *p* = ns		***Rs = 0.53; p = 0.04***
TLR4	Rs = 0.24; *p* = ns	**Rs = −0.62; *p* = 0.05**	Rs = 0.55; *p* = ns	

The results obtained for the gene expression data are showed in italics and the results obtained for the protein expression data are shown in regular font. In bold are highlighted the statistically significant results.

**Table 3:** Correlation between functional parameters and trigger gene and protein expression by clinical groups.

(A) Allergic asthmatic patients					
		**FEV1**	FVC	FEV/FVC	PBE
AKT1	Gene	Rs = −0.28; *p* = ns	Rs = −0.30; *p* = ns	Rs = −0.15; *p* = ns	Rs = 0.28; *p* = ns
	Protein	Rs = −0.59; *p* = ns	Rs = −0.62; *p* = ns	Rs = −0.16; *p* = ns	Rs = −0.16; *p* = ns
MAPK13	Gene	**Rs = −0.39; *p* = 0.009**	**Rs = −0.36; *p* = 0.02**	Rs = −0.13; *p* = ns	Rs = 0.07; *p* = ns
	Protein	Rs = 0.27; *p* = ns	Rs = 0.36; *p* = ns	Rs = −0.38; *p* = ns	Rs = −0.19; *p* = ns
STAT1	Gene	**Rs = −0.41; *p* = 0.006**	**Rs = −0.41; *p* = 0.005**	Rs = −0.25; *p* = ns	**Rs = 0.30; *p* = 0.04**
	Protein	**Rs = −0.63; *p* = 0.01**	Rs = −0.41; *p* = ns	Rs = −0.14; *p* = ns	Rs = 0.15; *p* = ns
TLR4	Gene	Rs = −0.19; *p* = ns	Rs = −0.23; *p* = ns	**Rs = −0.41; *p* = 0.009**	Rs = 0.15; *p* = ns
	Protein	**Rs = −0.62; *p* = 0.02**	**Rs = −0.62; *p* = 0.01**	Rs = 0.24; *p* = ns	Rs = 0.15; *p* = ns
(B) Nonallergic asthmatic patients					
		FEV1	FVC	FEV/FVC	PBE
AKT1	Gene	Rs = 0.24; *p* = ns	Rs = 0.45; *p* = ns	Rs = 0.39; *p* = ns	Rs = 0.06; *p* = ns
	Protein	Rs = −0.08; *p* = ns	Rs = 0.53; *p* = ns	Rs = −0.25; *p* = ns	Rs = 0.02; *p* = ns
MAPK13	Gene	Rs = 0.17; *p* = ns	Rs = 0.36; *p* = ns	**Rs = 0.49; *p* = 0.04**	Rs = 0.06; *p* = ns
	Protein	Rs = 0.04; *p* = ns	Rs = 0.57; *p* = ns	Rs = 0.19; *p* = ns	Rs = −0.24; *p* = ns
STAT1	Gene	Rs = 0.25; *p* = ns	**Rs = 0.46; *p* = ns**	Rs = 0.36; *p* = ns	Rs = 0.09; *p* = ns
	Protein	Rs = 0.43; *p* = ns	**Rs = 0.58; *p* = 0.04**	Rs = −0.06; *p* = ns	**Rs = −0.56; *p* = 0.04**
TLR4	Gene	Rs = −0.22; *p* = ns	Rs = 0.16; *p* = ns	Rs = −0.15; *p* = ns	Rs = −0.11; *p* = ns
	Protein	**Rs = 0.65; *p* = 0.03**	**Rs = 0.85; *p* = 0.002**	Rs = 0.20; *p* = ns	Rs = −0.13; *p* = ns

PBE: peripheral blood eosinophils (cells/mm^3^). In bold are highlighted the statistically significant results.

**Table 4:** Differential gene expression results in asthmatic patients and healthy control subjects for the main proteins of the signaling pathways of the four asthma triggers.

(A) Allergic asthma (AA) vs. Healthy Control (HC)										
	Gene	Gene Classification	Total AA		Severe AA		Moderate AA		Mild AA	
			RQ	Adj P	RQ	Adj P	RQ	Adj P	RQ	Adj P
AKT1 pathway	*AKT1*	Trigger	0.177	<0.001	0.109	<0.001	0.154	<0.001	0.354	ns
	*COL5A1*	Link Node	0.177	0.004	0.067	<0.001	0.203	ns	0.467	ns
	*MMP9*		0.041	<0.001	0.027	<0.001	0.033	0.006	0.082	ns
	*RELA*		0.165	<0.001	0.088	<0.001	0.154	<0.001	0.379	ns
	*SMAD3*		0.25	<0.001	0.134	<0.001	0.218	<0.001	0.616	ns
	*TLR2*		0.102	<0.001	0.051	<0.001	0.117	<0.001	0.177	ns
MAPK13 pathway	*MAPK13*	Trigger	0.177	<0.001	0.109	<0.001	0.154	<0.001	0.354	ns
	*IL12A*	Link Node	0.218	0.011	0.154	<0.001	0.165	0.018	0.435	ns
	*IL13RA1*		0.117	<0.001	0.072	<0.001	0.125	<0.001	0.177	0.029
	*NFATC1*		0.144	<0.001	0.067	<0.001	0.117	<0.001	0.5	ns
	*RELA*		0.165	<0.001	0.088	<0.001	0.154	<0.001	0.379	ns
STAT1 pathway	*STAT1*	Trigger	0.117	<0.001	0.063	<0.001	0.109	<0.001	0.287	ns
	*CCL5*	Link Node	0.145	<0.001	0.067	<0.001	0.127	<0.001	0.412	ns
	*COL5A1*		0.177	0.004	0.067	<0.001	0.203	ns	0.467	ns
	*IL10*		0.067	<0.001	0.038	<0.001	0.088	0.008	0.117	ns
	*IL12A*		0.218	0.011	0.154	0.001	0.165	0.018	0.435	ns
	*MMP9*		0.041	<0.001	0.027	<0.001	0.033	0.006	0.082	ns
	*RELA*		0.165	<0.001	0.088	<0.001	0.154	<0.001	0.379	ns
TLR4 pathway	*TLR4*	Trigger	0.072	<0.001	0.038	<0.001	0.072	<0.001	0.144	ns
	*MMP9*	Link Node	0.041	<0.001	0.027	<0.001	0.033	0.006	0.082	ns
	*RELA*		0.165	<0.001	0.088	<0.001	0.154	<0.001	0.379	ns
	*SMAD3*		0.250	<0.001	0.134	<0.001	0.218	<0.001	0.616	ns
Common	*ALOX5*	Mechanistic	0.102	<0.001	0.044	<0.001	0.088	<0.001	0.287	ns
	*BAX*		0.233	<0.001	0.154	<0.001	0.203	<0.001	0.5	ns
	*IL4R*		0.134	<0.001	0.063	<0.001	0.117	<0.001	0.435	ns
	*TGFB1*		0.180	<0.001	0.11	<0.001	0.157	<0.001	0.337	ns
(B) Nonallergic asthma (NA) vs. Healthy Control (HC)										
	Gene	Gene classification	Total NA		Severe NA		Moderate NA		Mild NA	
			RQ	Adj P	RQ	Adj P	RQ	Adj P	RQ	Adj P
AKT1 pathway	*AKT1*	Trigger	0.067	<0.001	0.072	0.006	0.067	<0.001	0.054	<0.001
	*CDH1*	Link Node	0.044	<0.001	0.077	ns	0.038	<0.001	0.036	<0.001
	*RELA*		0.062	<0.001	0.054	0.002	0.072	<0.001	0.058	<0.001
	*SMAD3*		0.125	<0.001	0.117	0.002	0.134	<0.001	0.109	<0.001
	*TLR2*		0.044	<0.001	0.027	0.002	0.036	<0.001	0.088	0.004
MAPK13 pathway	*MAPK13*	Trigger	0.036	<0.001	0.022	0.004	0.033	<0.001	0.058	<0.001
	*CXCL8*	Link Node	0.058	ns	0.054	ns	0.054	ns	0.072	ns
	*NFATC1*		0.067	<0.001	0.063	0.002	0.067	<0.001	0.077	<0.001
	*RELA*		0.062	<0.001	0.054	0.002	0.072	<0.001	0.058	<0.001
STAT1 pathway	*STAT1*	Trigger	0.051	<0.001	0.044	0.002	0.063	<0.001	0.047	<0.001
	*CXCR3*	Link Node	0.077	<0.001	0.088	0.019	0.077	<0.001	0.082	0.001
	*RELA*		0.062	<0.001	0.054	0.002	0.072	<0.001	0.058	<0.001
	*VCAN*		0.058	<0.001	0.041	0.002	0.054	<0.001	0.095	<0.001
TLR4 pathway	*TLR4*	Trigger	0.036	<0.001	0.022	0.004	0.033	<0.001	0.058	<0.001
	*CDH1*	Link Node	0.044	<0.001	0.077	ns	0.038	<0.001	0.036	<0.001
	*RELA*		0.062	<0.001	0.054	0.002	0.072	<0.001	0.058	<0.001
	*SMAD3*		0.125	<0.001	0.117	0.002	0.134	<0.001	0.109	<0.001
	*TLR5*		0.051	<0.001	0.102	ns	0.038	<0.001	0.054	0.002
Common	*ALOX5*	Mechanistic	0.038	<0.001	0.038	0.002	0.038	<0.001	0.036	<0.001
	*CCL5*		0.058	<0.001	0.051	0.002	0.063	<0.001	0.054	<0.001
	*PTGER2*		0.067	<0.001	0.036	0.002	0.077	<0.001	0.077	<0.001

RQ: Relative quantification with respect to HC. Ns: not statistically significant.

**Table 5:** Differential gene expression results between asthmatic patients (AA vs. NA) for the genes common in both clinical phenotypes groups by triggering pathways.

	Gene	Gene Classification	AA RQ	NA RQ	Adjusted P
AKT1 Pathway	*AKT1*	Trigger	0.177	0.067	0.008
	*RELA*	Link Node	0.165	0.062	<0.001
	*SMAD3*		0.250	0.125	0.021
	*TLR2*		0.102	0.044	0.022
MAPK13 Pathway	*MAPK13*	Trigger	0.177	0.036	0.021
	*NFATC1*	Link Node	0.144	0.067	0.028
	*RELA*		0.165	0.062	<0.001
STAT1 pathway	*STAT1*	Trigger	0.117	0.051	0.007
	*RELA*	Link Node	0.165	0.062	<0.001
TLR4 pathway	*TLR4*	Trigger	0.072	0.036	ns
	*RELA*	Link Node	0.165	0.062	<0.001
	*SMAD3*		0.250	0.125	0.021
Commons	*ALOX5*	Mechanistic	0.102	0.038	0.011

RQ: relative quantification of the gene expression value obtained in each asthma group compared to the control group. AA: allergic asthmatic patients; NA: nonallergic asthmatic patients. Ns: not significant.

**Table 6:** Genes studied, their classification in the signaling pathways of the *trigger proteins* in both asthma endotypes, chromosome location, and primers used for the gene expression analysis.

Gene	Definition of the Gene According to Biology Systems [12]	Chromosome Location (GRCh38/hg38)	Primer Reference
*AKT1*	Trigger	Chr.14: 104769349–104795743	Hs00178289_m1
*ALOX5*	Mechanistic in NA and AA	Chr.10: 45374166–45446121	Hs01095330_m1
*BAX*	Mechanistic in AA	Chr.19: 48954825–48961798	Hs00180269_m1
*CCL5*	Mechanistic in NA, link node in AA	Chr.17: 35871491–35880373	Hs00982282_m1
*CCL11*	Mechanistic in NA	Chr.17: 34285668–34288180	Hs00237013_m1
*CCL17*	Link node in NA	Chr.16: 57396076–57416063	Hs00171074_m1
*CDH1*	Link node in NA	Chr.16: 68737290–68835542	Hs01023895_m1
*COL5A1*	Link node in AA	Chr.9: 134641790–134844843	Hs00609088_m1
*CXCR3*	Link node in NA	Chr.X: 71615913–71618517	Hs01847760_s1
*IL4R*	Mechanistic in AA	Chr.16: 27313668–27364778	Hs00166237_m1
*IL8 (CXCL8)*	Link node in NA	Chr.4: 73740506–73743716	Hs00174103_m1
*IL10*	Link node in AA	Chr.1: 206767603–206772494	Hs00961622_m1
*IL12A*	Link node in AA	Chr.3: 159988836–159996019	Hs01073447_m1
*IL12B*	Link node in AA	Chr.5: 159314783–159330473	Hs01011518_m1
*IL13RA1*	Link node in AA	Chr.X: 118726954–118794533	Hs00609817_m1
*IL17A*	Mechanistic in NA and AA	Chr.6: 52186387–52190638	Hs00174383_m1
*MAPK13*	Trigger	Chr.6: 36130484–36144524	Hs00559623_m1
*MUC5B*	Mechanistic in NA and AA	Chr.11: 1223065–1262176	Hs00861595_m1
*MMP9*	Link node in AA	Chr.20: 46008908–46016561	Hs00957562_m1
*NFATC1*	Link node in NA and AA	Chr.18: 79395772–79529323	Hs00542678_m1
*PTGER2*	Mechanistic in NA	Chr.14: 52314298–52328606	Hs04183523_m1
*RELA*	Link node in NA and AA	Chr.11: 65653596–65662972	Hs00153294_m1
*SMAD3*	Link node in NA and AA	Chr.15: 67065698–67195195	Hs00969210_m1
*STAT1*	Trigger	Chr.2: 190969036–191014250	Hs01013996_m1
*TGFB1*	Mechanistic in AA	Chr.19: 41330531–41353933	Hs00998133_m1
*TLR2*	Link node in NA and AA	Chr.4: 153684080–153710643	Hs00152932_m1
*TLR4*	Trigger	Chr.9: 117704175–117717491	Hs00152939_m1
*TLR5*	Link node in NA	Chr.1: 223108401–223143282	Hs01920773_s1
*VCAN*	Link node in NA	Chr.5: 83471674–83582303	Hs00171642_m1
*18S*	Endogenous	-	Hs99999901_s1

In red are highlighted those genes studied but not detected in the samples analyzed.

## Data Availability

The data presented in this study are available on request from the corresponding author. The data are not publicly available as they are part of an ongoing study.

## Supplementary Materials

The following supporting information can be downloaded at: https://www.mdpi.com/article/10.3390/ijms26136240/s1.
