# Supplementary material for: Molecular Study from the Signaling Pathways of Four Potential asthma triggers: AKT1, MAPK13, STAT1, and TLR4
Source: Int J Mol Sci. 2025 Jun 28;26(13):6240. doi: 10.3390/ijms26136240 (PMC12249927; doi:10.3390/ijms26136240)
Supplement: Supplementary file 1 [file ijms-26-06240-s001.zip › ijms-3680874-supplementary.pdf]

International Journal of
Molecular Sciences

MDPI

Supplementary figures

Molecular Study from the Signaling Pathways of Four Potential *asthma triggers*: AKT1, MAPK13, STAT1, and TLR4

Lucía Cremades-Jimeno [1], María López-Ramos [1], Rubén Fernández-Santamaría [1], María Ángeles De Pedro [1], Ignacio Mahillo [2], **Cristina Rosales-Ariza [1], José María Olaguibel [3,4], Victoria del Pozo [1,4], María Luisa Caballero [4,5], Juan Alberto Luna-Porta [4,5], Santiago Quirce [4,5], Blanca Barroso [6], Diana Betancor [6], Marcela Valverde-Monge [6], Joaquín Sastre [4,6], Selene Baos [1] and Blanca Cárdaba [1,4,*]**

[1] Immunology Department, IIS-Fundación Jiménez Díaz-UAM, 28040 Madrid, Spain;
[2] Biostatistics and Epidemiology Unit, University Hospital Fundación Jiménez Díaz, 28040 Madrid, Spain;
[3] Allergology Service, University Hospital of Navarra, 31008 Pamplona, Spain;
[4] Ciber de Enfermedades Respiratorias (CIBERES), 28029 Madrid, Spain;
[5] Department of Allergy, La Paz University Hospital, IdiPAZ, 28029 Madrid, Spain
[6] Allergy Department, University Hospital Fundación Jiménez Díaz, 28040 Madrid, Spain
[*] Correspondence: bcardaba@fjd.es

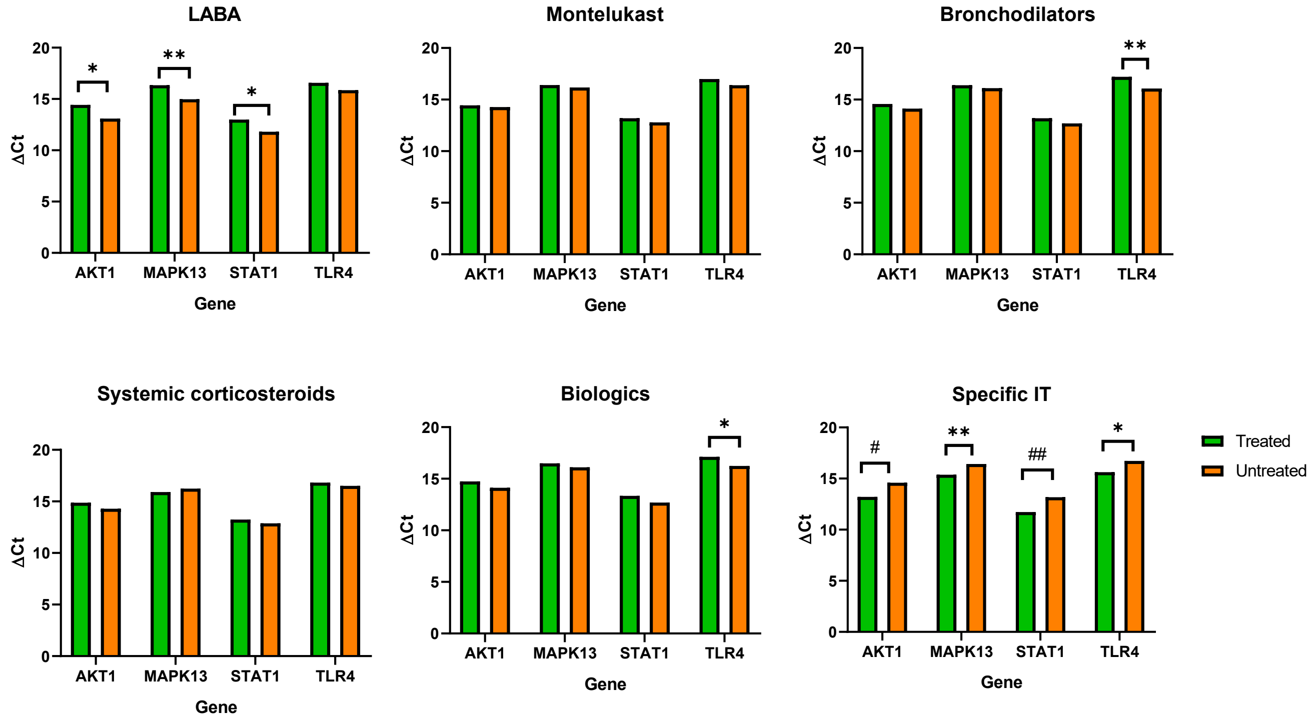

Figure S1. Differences in the gene expression of the four asthma triggers depending on the treatment of the patient. Gene expression results, showed as ΔCt, obtained in treated vs untreated patients for each different treatment. LABA: Long-acting beta-agonists; IT: immunotherapy. Statistically significant differences between treated and untreated patients are shown as: *, **, # and ## (p<0.05, p<0.01, p>0.005 and p<0.001, respectively).

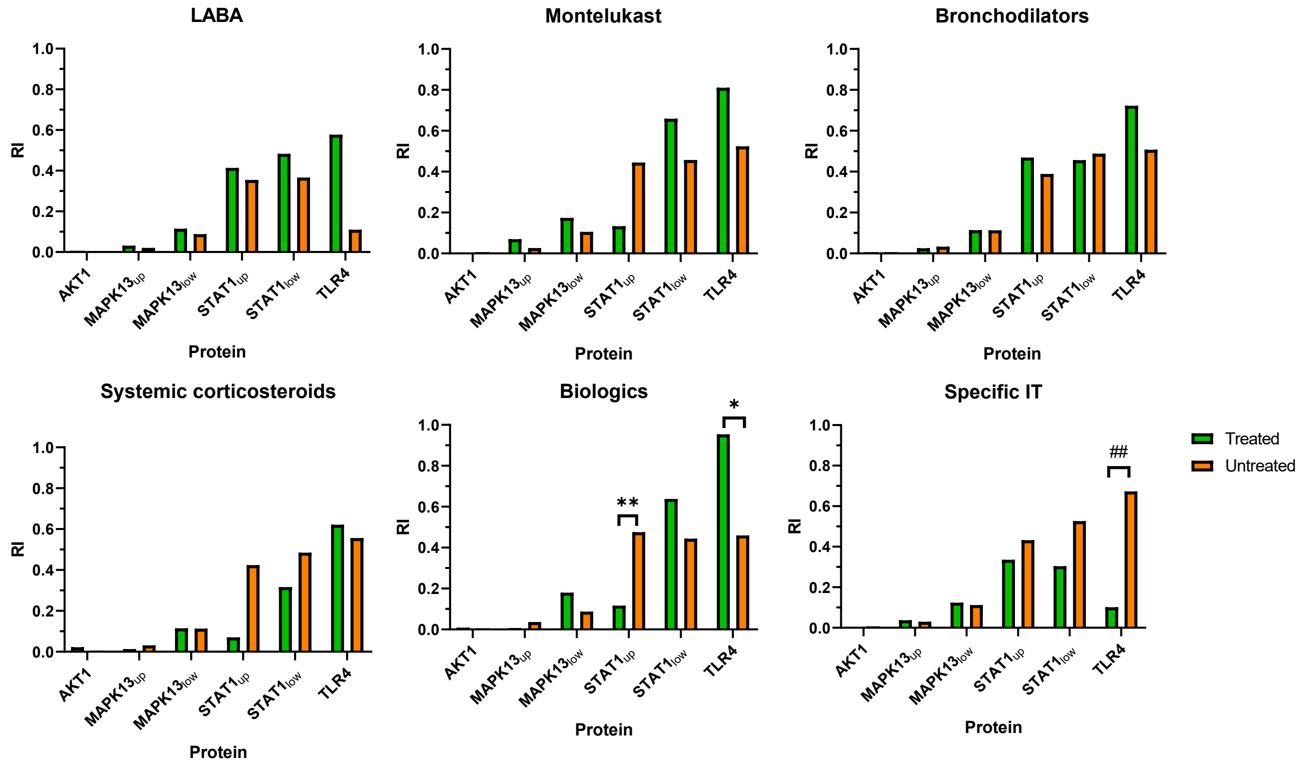

Figure S2. Differences in the protein expression of the four asthma triggers depending on the treatment of the patient. Protein expression results, showed as the relative intensity (RI), obtained in treated vs untreated patients for each different treatment. LABA: Long-acting beta-agonists; IT: immunotherapy. Statistically significant differences between treated and untreated patients are shown as: *, ** and ## ($p<0.05$, $p<0.01$ and $p<0.001$, respectively)
